# Settle Down! Ranging Behaviour Responses of Roe Deer to Different Capture and Release Methods

**DOI:** 10.3390/ani11113299

**Published:** 2021-11-18

**Authors:** Ulrika A. Bergvall, Nicolas Morellet, Petter Kjellander, Geir R. Rauset, Johannes De Groeve, Tomasz Borowik, Falko Brieger, Benedikt Gehr, Marco Heurich, A.J. Mark Hewison, Max Kröschel, Maryline Pellerin, Sonia Saïd, Leif Soennichsen, Peter Sunde, Francesca Cagnacci

**Affiliations:** 1 Grimsö Wildlife Research Station, Department of Ecology, Swedish University of Agricultural Sciences, 730 91 Riddarhyttan, Sweden; petter.kjellander@slu.se; 2 Université de Toulouse, INRAE, CEFS, 31326 Castanet-Tolosan, France; nicolas.morellet@inrae.fr (N.M.); mark.hewison@inrae.fr (A.J.M.H.); 3 LTSER ZA PYrénéesGARonne, 31320 Auzeville-Tolosane, France; 4 Terrestrial Ecology, Norwegian Institute for Nature Research (NINA), P.O. Box 5685 Torgarden, 7485 Trondheim, Norway; geir.rauset@nina.no; 5 Research and Innovation Centre, Biodiversity and Molecular Ecology Department, Fondazione Edmund Mach, Via Mach 1, 38010 San Michele all’Adige, Italy; j.degroeve@uva.nl (J.D.G.); francesca.cagnacci@fmach.it (F.C.); 6 Department of Geography, Ghent University, 9000 Ghent, Belgium; 7 Institute for Biodiversity and Ecosystem Dynamics, University of Amsterdam, 94240 Amsterdam, The Netherlands; 8 Mammal Research Institute, Polish Academy of Sciences, Stoczek, 17-230 Białowieża, Poland; tborowik@ibs.bialowieza.pl (T.B.); leif.soennichsen@gmx.de (L.S.); 9 Forest Research Institute Baden-Wuerttemberg, 79100 Freiburg, Germany; Falko.Brieger@Forst.bwl.de (F.B.); Max.Kroeschel@Forst.bwl.de (M.K.); 10 Department of Evolutionary Biology and Environmental Studies, University of Zurich, Winterthurerstrasse 190, CH-8057 Zurich, Switzerland; benedikt.gehr@ieu.uzh.ch; 11 Department of Visitor Management and National Park Monitoring, Bavarian Forest National Park, 94481 Grafenau, Germany; Marco.Heurich@npv-bw.bayern.de; 12 Wildlife Ecology and Wildlife Management, Faculty of Environment and Natural Resources, University of Freiburg, 79106 Freiburg, Germany; 13 Institute for Forest and Wildlife Management, Campus Evenstad, Innland Norway University of Applied Science, 2480 Koppang, Norway; 14 Office Français de la Biodiversité, Direction de la Recherche et de l’Appui Scientifique, 01330 Birieux, France; maryline.pellerin@ofb.gouv.fr (M.P.); sonia.said@ofb.gouv.fr (S.S.); 15 Department of Ecoscience, Aarhus University, Grenåvej 14, 8410 Rønde, Denmark; psu@ecos.au.dk

**Keywords:** box trap, *Capreolus capreolus*, net drives, net trap, roe deer, 3R’s

## Abstract

**Simple Summary:**

The study of animal movement in wild, free ranging species is fundamental for advancing knowledge on ecosystem relationships and for conservation. The deployment of bio-logging devices to this purpose (often GPS-collars in large mammals) requires relatively invasive procedures, such as capture, handling and release. Capture and manipulation cause behavioural modifications that are largely understudied in wild species and may affect both the welfare of animals and the output of the studies. We evaluated post capture and release ranging behaviour responses of a small deer species (roe deer *Capreolus capreolus*) for five different capture methods across 14 study sites within the EURODEER collaborative project. Roe deer showed modifications in their movement behaviour, independently of the capture method. However, individuals recovered rapidly, converging towards the average behaviour within a relatively short interval of time (between 10 days and one month), demonstrating a general resilience to such stressful events. We encourage researchers to continually adapt capture and handling methods so as to minimize stress and prioritize animal welfare.

**Abstract:**

The fitting of tracking devices to wild animals requires capture and handling which causes stress and can potentially cause injury, behavioural modifications that can affect animal welfare and the output of research. We evaluated post capture and release ranging behaviour responses of roe deer (*Capreolus capreolus*) for five different capture methods. We analysed the distance from the centre of gravity and between successive locations, using data from 14 different study sites within the EURODEER collaborative project. Independently of the capture method, we observed a shorter distance between successive locations and contextual shift away from the home range centre of gravity after the capture and release event. However, individuals converged towards the average behaviour within a relatively short space of time (between 10 days and one month). If researchers investigate questions based on the distance between successive locations of the home range, we recommend (1) initial investigation to establish when the animals start to behave normally again or (2) not using the first two to three weeks of data for their analysis. We also encourage researchers to continually adapt methods to minimize stress and prioritize animal welfare wherever possible, according to the Refinement of the Three R’s.

## 1. Introduction

Animal movements are shaped by evolutionary and ecological processes that can be researched through bio-logging [1]. While the study of animal movement is fundamental for conservation [2], the deployment of tracking devices on wild animals (often GPS collars on large mammals) requires relatively invasive procedures, such as capture, handling and release. Capture and manipulation cause stress, injuries, along with behavioural and physiological modifications that in some instances might be fatal [3,4]. As with other threatening events in an animals’ life, each capture and release induces physiological and psychological responses that have the potential to affect the animals’ behaviour for several days or weeks [4,5]. Although some authors have suggested that the understanding of the potentially deleterious effects of fitting these devices, including capture, handling and release, have not kept pace with the advances in the techniques themselves [6,7], several studies have addressed post capture and release behavioural responses. In particular, these studies focused on behaviours after the capture and release, such as grooming [8] physiological responses to the handling event [3,4], and ranging behaviour after capture [9,10]. Most of these studies showed a time-since-release effect in the behavioural metrics considered so that capture-and-release responses were hypothesised to be temporary, but in different ways between studies and species [9,10,11].

Capture and handling includes several stressful and physically demanding events that occur either at the same time or at consecutive points in time. Some parts of the capture and release event involve the presence of humans, others involve sudden or loud noises, while some parts involve social isolation or limited vision capability, which all represent different sources of stress [12,13]. Human presence is probably more stressful than the restraint itself [4,14], but this can be influenced by previous exposure to humans [15,16]. Additionally, novelty itself acts as a strong stressor, and therefore, induces fear reactions [17,18]. In addition, each capture and release session induces a range of physiological changes as a result of physical activity, such as running, struggling and transport [13], but also exhaustion for the emotional response to an intimidating stimulus [19]. Typically, the strength of these emotional reactions will be relative to the experienced fear for each event. Additionally, the reaction towards an event is dependent on what an animal has experienced in proximity to the event. Sensitisation (due to previous exposure to a stressor) can increase perceived fear and thus the reaction towards a stimulus [20]. Thus, the order in which events occur during a capture event can affect the strengths of the reactions. In turn, the order with which different stressful events occur differs between capture methods. Finally, if the capture protocol entails anaesthesia or sedation, individuals need to metabolise these compounds [21], which might influence movement capacity after capture and release.

According to the “Three R’s guidelines” for the ethical evaluation of animal use (Replacement, Reduction and Refinement; [22], capture-induced effects should be quantified to strive for minimisation of the negative impacts on animals [23,24]. Thus, the evaluation and improvement of capture methods should be a priority for researchers and conservation biologists. Buchanan et al. [25] state that investigators should consider the effects of capture, handling and marking of wild animals, and “select the least disruptive, as well as the least stressful, techniques available in the context of the study”. In addition to animal welfare and ethical considerations [3,4,26]; see also International Bio-Logging Society Constitution [27], the growing interest in capture effects is related to data reliability. For example, the study of animal movement and space use could potentially be biased by spurious effects due to capture disturbance [8,9,10,11].

Measuring the effect of capture and release operations on free-ranging animals is a non-trivial task though, because: (1) the pre-capture behaviour is normally unknown; (2) it is essentially impossible to have proper controls (but see [11]). Among the types of responses that can be recorded after release, those dependent on direct observation are normally not feasible in wild settings. Ranging behaviour, instead, can be directly measured by GPS devices often deployed on larger species at capture [9,10] and is intimately connected to vital activities, such as the acquisition of resources [28] and use of refuges and resting sites [29,30]. However, few studies of post capture ranging behaviour have accounted for different trapping methods and procedures (but see [31,32]). In the present study, we address this knowledge gap by focussing on the post capture and release ranging behaviour responses of 478 individual roe deer (*Capreolus capreolus*), using a wide range of capture methods. We measured two ranging behaviour metrics over time, namely distance between successive locations and distance from the centre of gravity, to evaluate the temporal nature of the behavioural responses to capture and release. We used these two metrics to quantify both the movement response (distance between successive locations) and the global effect on ranging behaviour (distance from the centre of gravity). By analysing the movement of individuals from different populations across Europe, we were able to compare behavioural responses to different capture methods, which comprise different sources of stress, and the order of exposure to different stressors (Table 1): box traps, with short and relatively long (one night) waiting time to release; net drives, with and without sedation; and dropping nets around an attractive point, or ‘net trap’ (See Methods and Appendix A for capture method description).

Because of the fear reaction to the capture event, we expected roe deer to move away from the capture site, which would likely result in an increase in distance from the centre of gravity. However, because roe deer tend to seek cover, increase vigilance, and decrease movement rate in the presence of risk (e.g., [33,34], and because of stress and exhaustion [19], we also predicted that roe deer would decrease their movements after capture. The return to the centre of gravity, though, is predicted to be more rapid than the recovery of average movement rate, as roe deer have a strong attraction towards familiar sites [28,30,35]. In general, the recovery time is expected to differ between capture methods which impose different sources of stress. Animals exposed to net-drives are expected to have a slower return to the centre of gravity since the disturbance is at the scale of the home range (Table 1). We also predicted that sedated animals would have a shorter distance between successive locations than animals handled without sedation, prior to the sedation effects wearing off [21]. For box traps, we expected that animals who spent a shorter amount of time in the trap prior to release would recover normal movement behaviour faster than animals exposed to the other box trap method [17]. If we take the order of events into account, we expected that animals that experienced the more frightening stressful event (namely, human presence) at the beginning of the capture event would show a more severe and prolonged reaction compared with those that experienced the more stressful event at the end of the capture process [20]. Thus, we expected that net drives and net trap methods would be more stressful and thus elicit a more marked response than the box trap method.

## 2. Materials and Methods

### 2.1. Study Species and Study Sites

The roe deer is a small (20–30 kg) solitary ruminant with a low level of sexual dimorphism, classified as a concentrate selector, which can form ephemeral feeding groups during winter [36]. Males are territorial and solitary in spring-summer [36]. It is characterised by high ecological flexibility (e.g., [37]), so that it can easily adapt to a variety of habitats across its distribution range in the European continent, from Scandinavia to the Mediterranean basin [38].

In this study, we investigated post-capture and release ranging behaviour for a total of 478 roe deer captured for their first time (226 fawns, 82 yearlings and 255 adults, where 85 of the individuals were followed over two age classes as they transitioned from fawn to yearling, or from yearling to adult) from 13 roe deer populations across Europe (EURODEER collaboration project, https://eurodeer.org (accessed on 15 October 2021); Table 1).

### 2.2. Capture Methods

Our sampled roe deer were captured with three main methods: box traps, net drives and net traps (Table 2). These can also be categorised in terms of the order of events, where box trap methods represent those with the most stressful events involving direct human contact at the end of the capture, while the net methods are those with the most stressful events occurring at the beginning of the process. Additionally, the methods may differ in terms of the time lag between capture and release. The methods are summarised below for the general and common procedures, while site-specific descriptions and details are provided in Appendix A Roe deer captures were performed during winter- and early-spring, when roe deer are less solitary, and before the territorial behaviour and the reproductive phase begins, normally in mid-April [36]. Specifically, we used capture events that occurred between 3rd October and 25th March, to allow at least 20 days of monitoring before 15th April (see below).

Box trap methods—Box traps are essentially wooden boxes with a fixed door, to which a small feeder may be attached, with a hanging door that is released by a mechanic trigger, which in turn is activated by touching or breaking a string or fishing line attached to the fixed end of the box. To retrieve the animal, the door is opened, one person restrains the back legs and pulls the animal out of the trap while a second person restrains the front legs. Typically, box traps are commonly used in areas with severe winters, where roe deer are attracted by the supplemental feeding provided in the trap. Often, traps are also paired with actual feeding stations that are active throughout winter [39].

We monitored 198 total capture events with two different box trap methods: “box trap (174 captures from seven populations), where the traps were checked several hours after capture (typically in the morning, if traps were set in the evening; [40,41]; “box trap short” (24 captures from two populations), when the traps were checked a maximum of two hours after capture that was notified to operators via remote communication [42].

Net drive methods—Net drives were deployed in strategic locations so as to encompass an area of woods or open areas, often leaving ‘one side’ open that could be walked by a line of beaters that move within sight distance of each other. Animals were ‘pushed forward’ by the constant moving line of beaters, towards the vertical nets. The net line was normally prepared beforehand to prioritize capture effectiveness and animal safety, e.g., removing branches or impediments that would prevent the nets falling properly, and avoiding risky areas (e.g., escarpments or cliffs). The nets were hung onto branches, trees or poles by thin nails or ‘V’ cuts in the poles to ensure that the nets dropped to the ground as soon as the animal entered one. Experienced operators waited nearby the nets to immediately restrain a captured animal, disentangle it, and proceed with the successive phases of the capture procedure that might or might not include sedation, and might or might not use a transportation box prior to marking (see Appendix A or variation in the protocols for the net drive method).

We monitored a total of 260 capture events with two different net drive methods: “net drives” (118 captures from six populations), and “net drives sedation” (142 captures from two populations), with the use of chemical sedation, specifically, with an intramuscular injection of acepromazine (calmivet 3cc). This is a short-acting neuroleptic that rapidly reduces the stress response and prevents adverse reactions in roe deer [43], but does not require an antagonist to reverse its rather short-term effects.

Net trap method—Net traps were usually mounted in the proximity of an attractive resource (typically feeding sites), where one or several individuals gathered to feed. Nets were either concealed on the ground or hung from poles. When animals were within their perimeter, the net traps were released (either being lifted or falling) to catch the animals. The release was either automatic, e.g., when animals stepped on a trigger or activated by operators watching nearby (e.g., with a CCTV). In either case, capture personnel immediately restrained the captured animals, disentangled them, and proceeded with the successive phases. We sampled 20 captures from three populations with the net trap method. Animals were sedated (*n* = 16) or not (*n* = 4).

### 2.3. Manipulation and Marking

When animals were extracted from nets and/or box traps, they were in some cases, but not all, fitted with a mask to decrease visual stress, and restrained by two or three operators, while a third/fourth one proceeded with the marking and measurement/sample collection.

Animals were fitted with a GPS-collar as age (three age classes: fawn, yearling, or adult), determined from body morphology and tooth eruption and wear features; [44] and body mass were considered compatible with the weight of the collar. The roe deer age was set to change on 1 April, so animals caught as fawns could transition into yearlings, or yearlings into adults, while followed for post-release behaviour.

In addition, a number of parameters were normally recorded during manipulation (including biometric measures like body temperature and body mass), samples taken (including tissue, blood, or hair for genetic analysis), and ear tags for visual recognition fitted too. These parameters were taken independently of the capture protocol (including the use of sedation).

### 2.4. Capture and Release Response Metrics and Statistics

Response after capture and release—We used two metrics to describe the ranging behaviour of roe deer after the capture and release: (i) the distance of successive locations from their centre of gravity, calculated from the date of capture to 60 days after capture, to estimate the number of days it took for the average distance to the centre of gravity to plateau; and (ii) the distance between successive locations, to estimate the time to settle after the capture and release event. Clearly, these are relative measures, as the baseline behaviour before capture is unknown.

The centre of gravity was determined between the date of capture, and the 15th of April of the same capture season; similarly, the distance between successive locations was measured between the date of capture and the 15 April of the same capture season. We retained individuals with a minimum of 20 days of monitoring in total and with the first location recorded within three days of the capture event. Because the sampling regime of GPS locations differed among and within study sites, we selected a consistent number of locations per unit of time for each individual. Specifically, we retained a maximum of six locations per 24 h, that is, at most, one fix every four hours, and with a maximum gap of three days (72 h) between successive fixes, as we did not interpolate missing data. Using these criteria, a total of 478 capture and release events and 113,138 locations were used for the analysis.

As individual variability in the use of space could confound the effect of the capture and release response on ranging behaviour, we controlled for the home range size in our analyses by adding the logarithm of the home range size as a fixed effect in the models. To do so, we estimated the home range size based on the locations between the 11th and the 21st day after capture, with ad hoc smoothing parameters for each individual home range (adehabitatHR package; [45] at the 90% isopleth [46]. Individuals with less than ten locations during this time frame were excluded.

### 2.5. Statistics

We used General Additive Mixed Models (GAMM) of the log-transformed (i) distance to the centre of gravity of all locations and (ii) distance between successive locations, with individual identity and study areas as random factors to control for repeated observations. Our baseline model included capture method (five methods), sex (male, female) and age at capture (fawn, yearling or adult) and the two-way interaction between sex, age, and method to account for possible method-, sex-, and age-related changes in ranging behaviour due to the capture event. Finally, we included home range size as a covariate to control for the potential influence of inter-individual variation in ranging extent. We used GAMM rather than standard linear models because GAMMs can more efficiently capture nonlinear temporal variation. Apart from the time spline, we did not specifically take into account the temporal autocorrelation between successive GPS locations of the same individual. To describe the post-capture pattern of temporal variation in the two metrics, we modelled a smoothed effect of time since capture, based on thin plate regression splines, which differed among capture methods. We used Akaike’s Information Criterion corrected for small sample size (AICc), AIC weights (AICcWt) and the number of parameters to select the most parsimonious model that best described the data [47]. We fitted models with the ‘gamm4′ function in the ‘gamm4 R package [48].

Based on the selected models for the two movement metrics, we estimated the number of days it took for roe deer to attain the average distance to the centre of gravity and the distance between successive locations for each capture method. The source data are archived in the Eurodeer/Euromammals dataset, accessible upon login. The source data file used for the analysis presented in this ms will be deposited in Zenodo upon acceptance.

## 3. Results

**Figure A1:**
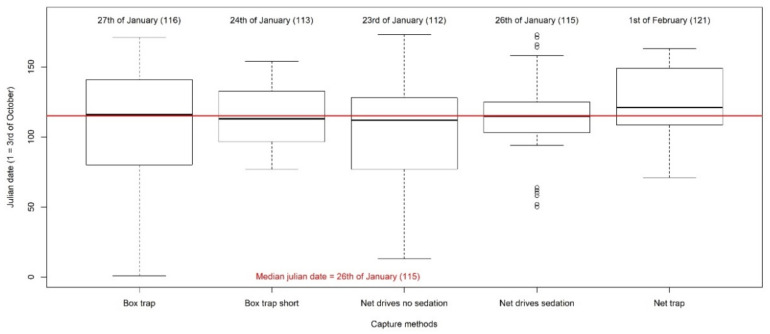
Date of capture and release for the five capture methods.

**Figure A2:**
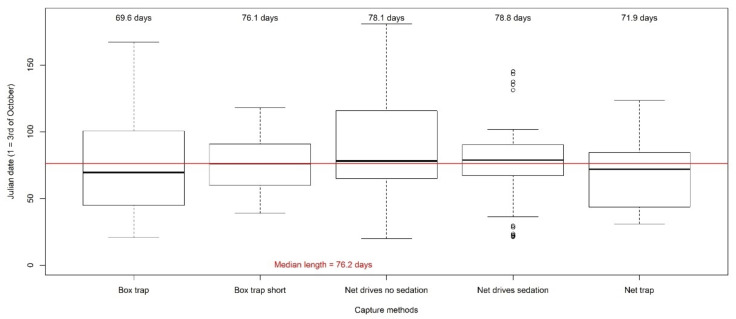
Duration of the sampling of roe deer from capture date to the 15 April for the five capture methods.

The median Julian date for captures across methods was 26 January (Figure A1), hence the duration of the sampling to evaluate the range behaviour metrics from capture to 15 April (last date of sampling before the reproductive season) was a median of 76.2 days (Figure A2).

**Figure 1:**
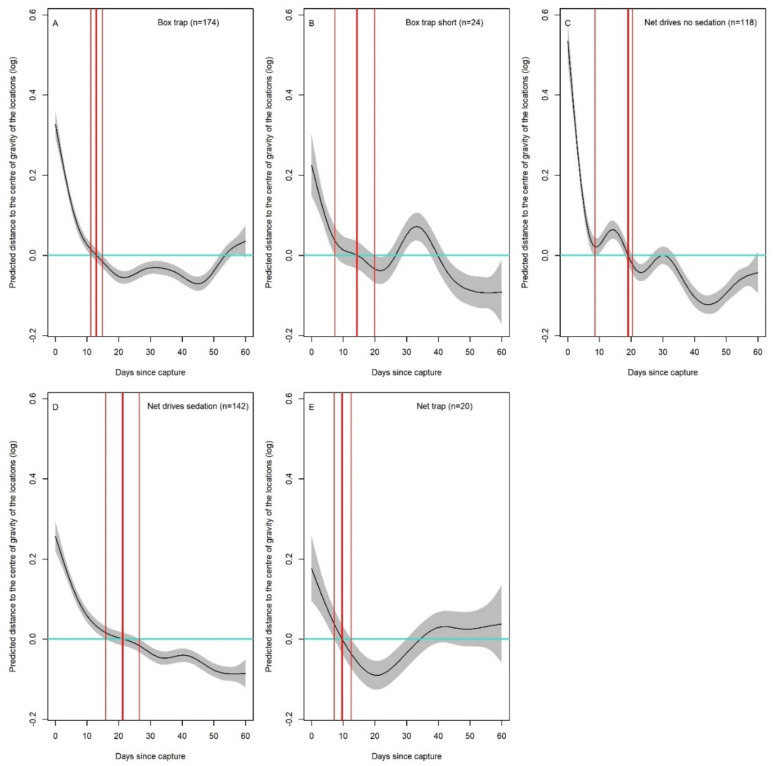
(**A**–**E**). Number of days after capture and release to rebound to the individual-specific average log-transformed distance to the centre of gravity of locations (thick red vertical line), the average distance +SE and the average distance −SE (thin vertical lines), across the different capture methods. The model is centred on zero, i.e., the plotted values sum to zero that hence represents the average distance (blue line: no effect). When the line crosses zero, it implies the distance has ‘rebounded to the overall average value.

**Figure A3:**
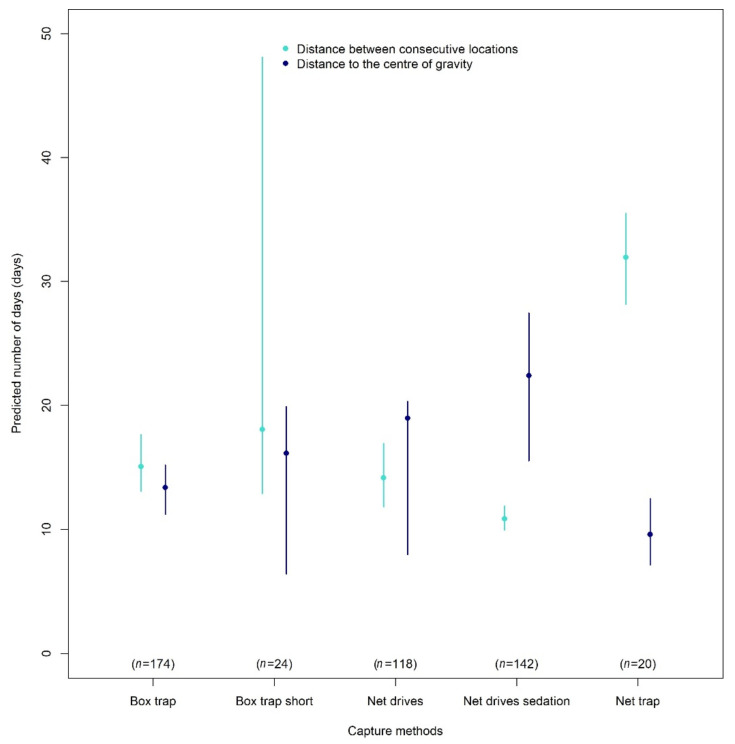
Recovery time for distance to centre of gravity, and between successive locations, for the five roe deer capture methods.

The most parsimonious model describing variation in the distance to the centre of gravity of locations (AICcWt = 0.34) included the spline of days since capture, which differed among capture methods, while controlling for the log-transformed home range size (Table S2). After capture and release, roe deer were located further from the centre of gravity of locations, but this distance decreased and stabilised, on average, around 20 days or less after capture for all methods (Figure 1 and Figure A3). Recovery time was shortest for “net trap” (9.6 (7.1–12.5); Figure 1E, slightly longer for “box trap” (13.4 (11.2–15.2); Figure 1A) and “box trap short” (16.2 (6.4–19.9); Figure 1B), and highest for “net drives” (19 (8–20.3); Figure 1C) and “net drives sedation” 22.4 (15.6–27.4)). The capture method, however, did not influence the absolute displacement away from the centre of gravity following capture and release, as capture method did not additionally contribute as a fixed factor to explain its variation. As expected, individuals living in a larger home range moved further, on average, as the distance to the centre of gravity of locations increased with the log-transformed home range size (slope ± SE = 0.284 ± 0.0254; Table S1).

**Figure 2:**
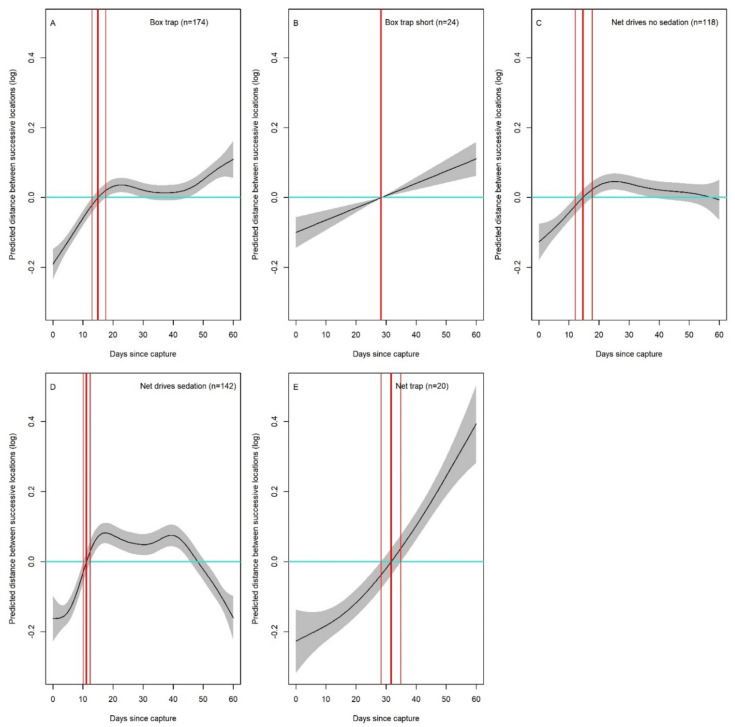
(**A**–**E**). Number of days after first capture and release to rebound to the average individual-specific distance (thick, red vertical line), the average distance +SE and the average distance −SE (thin red vertical lines) between successive locations, across the different capture methods. Light blue line: no effect (average distance). The model is centred on zero, i.e., the plotted value sum to zero that hence represents the average distance (blue line: no effect). When the line crosses zero, it implies the distance has ‘rebounded to overall average values.

The most parsimonious model describing variation in the distance between successive locations (AICcWt = 0.130) included the spline of days since capture, which differed among capture methods, while also controlling for the log-transformed home range size (Table S2). The capture method, however, did not influence the absolute distance between successive locations, as the capture method did not additionally contribute as a fixed factor to explain its variation. In general, the distance between successive locations was shorter than average immediately after the capture event, before increasing over time (Figure 2 and Figure A3). For “net drives sedation”, “box trap”, and “net drives” the recovery to the average distance between successive locations was a maximum of 15 days on average: 10.9 (10.0–11.9), 15.1 (13.1–17.7), and 14.2 (11.8–16.9) (Figure 2A,C,D). In contrast, recovery time was longer for “box trap short” and “net trap”, with 18.1 (12.9–48.1) and 32 (28.2–35.5) days, respectively (Figure 2B,E). Finally, the distance between successive locations also increased with the log-transformed home range size (slope ± SE = 0.131 ± 0.015; Table S2).

## 4. Discussion

Our large-scale analysis of the behavioural response of roe deer to first capture and release, in terms of movement and space use, showed a consistent re shift away from the centre of gravity and reduction in movement amplitude across all capture methods, confirming our general prediction (Table 2). For all capture and handling methods, roe deer recovered to average ranging behaviour in less than a month. Hence, in terms of behavioural impact, it was not possible to identify “the best overall method”, contrary to our expectations. The difference in recovery times for the two metrics over the different methods, though, seems to indicate that the order of events, rather than the means of physical capture in itself, affects the behavioural response (Figure A3). For example, “box trap short” has a longer recovery time for the amplitude of movements than “box trap”; similarly, “net trap” has a longer recovery time for the amplitude of movements than “net drives”, but a shorter recovery time for a return to the centre of gravity.

Previous studies have shown that ungulates have a long-term memory of negative experiences [12]. Hence, one way to assess an animals’ perception of handling and restraint is to measure its willingness to re-enter the specific area where it was first handled [49]. The use of this area may, therefore, be a proxy for how aversive a particular method was perceived. Methods that used a focal capture location that provided food, such as box traps and net traps, generated a fast recovery of average range use (distance from centre of gravity rebounded to its average in less than 15 days). In contrast, in line with our expectations, methods that created a disturbance over a larger spatial scale, such as net drives, provoked a more prolonged shift in the use of space. We attribute this difference both to how roe deer may link space to the risk associated with a given location (i.e., attribute memory vs. reference memory, [50] and to the type of disturbance. Net drives resemble a chase by hunters/predators that have been shown to stimulate a risk avoidance response in terms of space use in roe deer, thus pushing them to the edge of their home range [30,33,51]. Another factor affecting the perception of risk associated with the capture location is the potential positive reinforcement linked to it [20]. Indeed, box traps are often installed at supplemental feeding sites. In areas with substantial snow cover during winter and, thus, seasonally limited resource availability, the presence of food can significantly alter the ranging behaviour and timing of visitation of roe deer [42,52]. A novel object, such as the box trap is likely to initially induce fear [17] and a neophobic stress response (see [53] for an experimental approach on roe deer). Subsequently, however, when food is offered in proximity to and inside the boxes, habituation and counter conditioning should occur, so that the box will become a positive stimulus associated with food [41]. Hence, despite the stressful event of capture and release, may still be associated with a reward that encourages the animal to re-visit the area with the box trap independent from their willingness to re-enter the trap. Additionally, roe deer often form temporary feeding groups during winter so that naïve individuals may encourage previously caught animals to re-visit the feeding sites through social enhancement [54].

Increased movement has previously been related to higher predation risk in elk [55,56], while reduced movement rate in reaction to high hunting pressure has been recorded in roe deer [34]. Contrary to our expectations, recovery of average movement rate (i.e., the distance between successive locations) for net drives was as fast as for box traps but much shorter than for net traps. Although both net drives and net traps require the extraction and manipulation of the animals from nets, they differ in terms of the order of events. In a net trap, the net entanglement and manipulation by humans occur as a sudden and completely unexpected event, i.e., with no previous cues. For net drives, the first event that potentially provokes fear is the perception of an ‘approaching risk’ (the drive), which may last for a relatively prolonged period of time. The most stressful component, i.e., the presence of humans [4,14], occurs next, but the earlier presence of cues signalling a risky situation might allow a more gradual rise in the stress level, possibly priming the animal to cope with the moment of peak stress [17]. Similarly, for box traps, roe deer appeared to recover their average movement rate faster when there was a longer delay between capture and handling (e.g., through the night) than when they were handled almost immediately, supporting the ‘sensitisation’ hypothesis. When a roe deer is captured in a box trap, the two major events inducing stress are: first, when the box door closes so that hearing and sight are impaired; second, when humans arrive at the trap site. We speculate that the two stressful events (being caught and being manipulated) could be perceived as ‘distinct’, with no severe sensitisation, or a cumulative effect of stress [20] if a long delay between the two occurs. In contrast, if the roe deer has not yet acclimated to the first event (being caught in the trap) when humans arrive, the response to handling is likely more intense.

For example, domesticated animals are usually stressed during road transport in cattle trucks [57], but several studies on both wild and domesticated ungulates have shown that, in general, they cope with handling and transport [19,58]. However, animals show an increased reaction to truck transport if they first experienced a more threatening event [59].

Finally, overall, sedation did not attenuate responses to capture for net drives. The need to metabolize the sedatives [21] could result in a longer recovery. For example, the clinical effects of acepromazine can be present up to 48 h in older animals [21]. This could explain the longer time delay for roe deer that had been sedated at capture to rebound to the average distance between successive locations. We expected sedation to decrease the intensity of struggling and the impact of external stimulus substantially, thus reducing stress [21], but found little support for this. Further evaluations should probably be based on the comparison of physiological parameters, e.g., heartbeat, temperature, cortisol and lactic acid levels [60,61]. During severe stress, stimulation of the sympathetic nervous system induces physiological responses that can cause physiological changes, such as alterations in blood flow and depletion of normal aerobic energy, decreasing oxygen and nutrient supply to tissues [62].

Although stress responses have been found to be affected by age, sex, season, and stressor type in a variety of species [63,64,65,66,67], we found no support for sex- or age-related differences in the behavioural response to capture for roe deer.

Tame red deer (*Cervus elaphus*) decreased their grazing activity by 40% for the first eight days after being fitted with a collar [68]. Similarly, chemically immobilized Alpine ibex (*Capra ibex*) showed decreased movement rates for the first two days after the capture event [69], while this lasted for three to six weeks in Ursids [5]. Our results suggest that, in roe deer, location data collected with GPS collars reliably reflect “normal” behaviour only after a few weeks (two weeks for most methods, one month maximum for net traps and box traps with the immediate intervention of humans) that should, however, be treated with caution in movement analyses.

When researchers and conservation biologists choose methods, many factors are considered as practicality, accuracy, risk of biased results and ethical considerations. We urge the need for transparency of how researchers use the GPS positions directly after the capture event. If researchers investigate questions related to the distance between successive locations of the home range we recommend to, (1) test when the animals start to behave normal again or (2) not use the first two to three weeks for their analysis. We also encourage researchers to always strive to keep the capture and handling as good for the animals as possible and researchers should always strive to increase welfare according to the “Refinement” in the “Three R’s” [23]. In particular, we urge to consider studies like the present one to (i) a priori identify the order of events of different capture protocols, and (ii) to possibly quantitatively evaluate their actual provoked response in the study species.

## Figures and Tables

**Table 1:** Locations and methods of capture used across the study sites.

Study Site	Country	Location	Area	Method/s and Number of Individuals in Parenthesis
Aurignac ^1^	France	43°13′ N, 0°52′ E	75 km^2^	Net drives (73), Net drives sedation (116)
BavarianNP ^2^	Germany	49°83′ N, 13°81′ E	~1000 km^2^	Box trap (108)
Bernese ^3^	Switzerland	46˚55′ N, 7˚51′ E	~1500 km^2^	Box trap (14), net drives sedation (26)
BialowiezaNP ^4^	Poland	52°43′ N, 23°27′ E	175 km^2^	Net trap (16)
Bogesund ^5^	Sweden	59˚24′ N, 18˚12′ E	13 km^2^	Box trap (1)
Chize ^6^	France	46°05′ N, 0°25′ W	26.14 km^2^	Net drives (1)
Giudicarie ^7^	Italy	46°4′ N, 10°43′ E	230 km^2^	Box trap short (23) Net trap (1)
Grimsö ^5^	Sweden	59°40′ N, 15°25′ E	130 km^2^	Box trap (3)
Hegau Baden ^8^	Germany	47°50′ N, 8°43′ E	75 km^2^	Box trap (12)
Kalø ^9^	Denmark	56°17′ N, 10°29′ E	10 km^2^	Net drives (4)
Koberg ^10^	Sweden	58˚15′ N, 12˚44′ E	84 km^2^	Box trap (18)
Monte Bondone ^11^	Italy	46°1′ N, 11°2′ E	110 km^2^	Box trap short (1), Net drives (17)
Rhine Baden ^12^	Germany	48°38′ N, 7°59′ E	100 km^2^	Box trap (18), Net drives (9), Net trap (3)
Trois-Fontaines ^13^	France	48°43′ N, 4°56′ E	13.60 km^2^	Net drives (14)

^1^ All capture and marking procedures were done in accordance with French and European laws for animal welfare (prefectural order from the Toulouse Administrative Authority to capture and monitor wild roe deer and agreement no. A31113001 approved by the Departmental Authority of Population Protection). ^2^ The research program in the Bavarian Forest is managed by the Administration of the Bavarian Forest National Park. Game captures were conducted in accordance with European and German animal welfare laws. The experiment was designed to minimize animal stress and handling time, and to ensure animal welfare, as defined in the guidelines for the ethical use of animals in research. Animal captures and experimental procedures were approved by the Ethics Committee of the Government of Upper Bavaria and fulfils their ethical requirements for research on wild animals (Reference number 55.2-1-54-2531-82-10). ^3^ The animal capture and handling protocols were authorized by the cantonal veterinary and animal welfare services with permit number BE75/11. ^4^ Permissions for sampling of live animals were obtained from the Polish Ministry of Environment (Permit No. DLOPik-L-gl-6713/86b/07/ab) and the Local Ethical Commission in Białystok (Resolution No. 46/2008). ^5^ Ethical permission from Uppsala Board for Laboratory animals (Dnr: C149/2015). ^6,13^ All methods were approved by the authorities (French Ministry of Environment). Roe deer captures were performed in accordance with the conditions detailed in the specific accreditation delivered to the Office National de la Chasse by the Préfecture de Paris (agreement n°2009–014). ^7,11^ Approval for capture and marking by the Provincial government statements 18 September 2004 and 20 November 2011, under Ethical assessments of the Wildlife Committee of the Autonomous Province of Trento. ^8,12^. All capture, tagging and monitoring protocols were approved by the animal welfare and hunting administration of Baden-Wuerttemberg, Germany. ^9^ General institutional permission for capturing and marking birds and mammals, issued by the Danish Nature and Forest Agency (reference: SM 302-009). ^10^ Ethical permission from Gothenburg Board for Laboratory animals (Dnr: 405-2008).

**Table 2:** Brief description of capture methods and related predictions for the capture and release metrics (distance between successive locations, and distance to the centre of gravity of locations).

Method	Brief Description	Length of the Events	Order	Prediction	Brief Description
All protocols	-			Lower than average/Slower recovery time than centre of gravity	Higher than average/Faster recovery time (site-fidelity)
Box trap	Habituated to boxes, long acclimation time, medium increase in threat level (human arrival)	Long time (waiting in the closed trap) + short time (manipulation by humans)	Less stressful event first	Slower recovery time (long exposure)/alternatively	-
Faster recovery time (habituation)	Faster recovery time (focal-based disturbance)	-			
Box trap short	Habituated to boxes, short acclimation time, medium increase in threat level (human arrival)	Short time (waiting) + short time (manipulation)	Less stressful event first	Faster recovery time (short exposure)/alternatively Slower recovery time (sensitization)	Faster recovery time (focal-based disturbance)
Net drives no sedation	No acclimation, medium-delay cues in threat level increase	Long time (oncoming threat) + short time (manipulation by humans)	Most stressful event first	Slower recovery time (more stressful, frightening event first)	Slower recovery due to area avoidance (range-based disturbance)
Net drives sedation	No acclimation, medium-delay cues in threat level increase	Long time (oncoming threat) + long time (manipulation and chemical recovery)	Most stressful event first	Slower recovery time (more stressful, frightening event first)	Slower recovery due to area avoidance (range-based disturbance)

## Data Availability

The source data are archived in the Eurodeer/Euromammals dataset, accessible upon login. The source data file used for the analysis presented in this ms will be deposited in Zenodo upon acceptance.

## Supplementary Materials

The following are available online at https://www.mdpi.com/article/10.3390/ani11113299/s1, Figure S1: Model selection to access capture and release responses in roe deer in terms of their ranging behaviour, Table S1: Model selection table for the distance to the centre of gravity of the locations after capture and release (GAM of the log-transformed distance with individual identity and study area as random effects), Table S2: Model selection table for the distance between successive locations after first capture and release (GAM of the log-transformed distance with individual identity and study area as random effects).

## Appendix A

### Area Specific Description of Capture Methods

For the Aurignac study site, roe deer have been caught during annual winter captures since 1996. Due to the openness of the landscape, net drives capture involved large-scale drives with 30–100 beaters and up to 4 km of long-nets. Roe deer were driven for a variable period lasting generally less than 10 min. Once a deer was captured, it was removed from the net and transferred to a wooden retention box providing darkness and ventilation until the marking procedure (see [9] for more details). Most captured roe deer were tranquilized with an intramuscular injection of acepromazine (calmivet 3cc). Marking procedures lasted for approximately 10 min. and were performed by trained and experienced handlers. All animals were then released on site.

In the Bavarian Forest, roe deer are captured in wooden box traps at about 25 different locations with one or two traps at each trap site. The animals were lured with apple pomace and corn. The box-traps were typically prepared for trapping during sun set, between 15.00 and 17.00 and animals were released between 07.00 and 10.00 in the next morning. The handling normally involves two to four humans. The animals were held by one person by the legs and the eyes of the animal were covered by a blanket. Afterward, the total length and weight were measured and the collar fitted. No chemical immobilization or tranquillizer were used [70].

In the Bernese area, individual roe deer were captured in box traps and net drives. All the box traps (*n* = 10) were equipped with a sensor that sent an alarm to a researcher when an animal set off the trigger, which allowed to keep the traps activated 24 h a day. The response time after a trap alarm was typically several hours depending on the time of capture. A typical capture session using net drives involved between four to six helpers. The actual drive hunt usually lasted between a few minutes to half an hour. Captured animals were sedated (with acepromazine) upon capture and kept in a box for up to one hour to let the drug take effect and to let them recover from the capture event before handling.

In the western edges of the Białowieża Primeval Forest roe deer were trapped with drop-down nets baited with maize and hay. The drop-down net traps were baited for several weeks before the trapping event took place. Once a trap was regularly visited by roe deer it was prepared for trapping. After setting the trap it was surveyed by a camera and released manually. Researchers were on site within 60 s after releasing the net. In order to immobilise captured roe deer, we administered an intramuscular injection of a mixture (Hellabrunn mixture) of xylazine (Rompun©, Bayer Animal Health, Shawnee, KS, USA) and ketamine hydrochloride. Atipemazole hydrochloride (Antisedan^®^ Orion Corporation, Esboo, Finland) was used as an antidote [21]. Roe deer were equipped with GPS collar but no ear tags.

In the Grimsö, Bogesund, Koberg areas, roe deer have been caught during annual winter captures since 1973, 1988 and 2006 respectively. In each of the three areas, roe deer are captured in box traps at about 16 different locations with one to three traps at each trap site. The box traps were typically prepared for trapping during sunset, between 15.00 and 17.00 and animals were released between 07.00 and 10.00 in the next morning [41]. The handling normally involves 2 to 4 humans. No chemical immobilization or tranquillizer was used.

In Giudicarie-Rendena, roe deer were captured with wooden box traps, baited with pellets of cereals and corn, and placed in the proximity of feeding stations filled up ad libitum throughout the winter. Capture sessions lasted a few days each, during which access to the food in the feeding stations was prevented in order to drive the roe deer into the box traps. The box-trap mechanic closure trigger would (1) close the door (2) switch on a modem that in turn would send a warning to capture operators. This way, animals could be extracted, manipulated, and released a maximum of 2 h later than the trap shut. No sedation, nor tranquilizer chemicals were used. The manipulation lasted a maximum of 7 min, with a suite of biometric measures and sampling taken as in the general description, but usually no body-weight, given the difficult terrain where capture operations happened.

In Hegau and Rhine-Baden roe deer were caught in wooden box traps, net drives and net traps. The box traps were baited with apple pomace. The trigger of the trap was activated once the animals started to enter the trap. The status of the trap was monitored with a trap alarm that sent hourly SMS once the trap was released. A released trap (usually during night) was controlled the following morning at sunrise. Roe deer were handled by one operator without sedation while a second operator took measurements and attached the collar. Handling of the animals lasted a maximum of 10 min. Net traps consisted of a circular net that was hidden in the ground. The lower part of the net was attached to the ground while the upper part was pulled up with a counterweight after release. Animals were baited in the middle of the trap with apple pomace. The trapping team set up once the animals regularly entered the trap and constantly surveyed the trap with a camera from a distance. The trap was manually activated once the animals were inside the trap. Caught animals were released from the net and handled as with box traps. Net drives were usually carried out with more than 20 people. Once the area (field or forest patch that provided cover) was encircled with nets, most people gathered alongside the net while some went inside and tried to beat the animals into the net. When a deer was caught, the drive was paused and the deer was immediately removed from the net and placed in a small, darkened wooden box. It stayed there until the drive was finished (usually 15–30 min). Deer were not sedated and afterward handled as with box traps. In Hegau only box traps were used.

In Kalø net drives were usually deployed within decayed “nurse fences” (0.5–2 ha fenced areas used in the forest industry to protect younger trees from roe deer). When holes in the fences (1.5–1.8 m height, 2 mm metal wire with 20 × 20 cm mesh widths) appear after 5–15 years and the tree vegetation has reached a height of 2–8 m roe deer often stay within the fences at daytime. Capture attempts within such fences had the benefit of dense vegetation cover (1–6 m horizontal sight) around the nets, limited escape possibilities after entrance holes were blocked and because the fences themselves sometimes functioned as nets. In cases of such limited escape possibilities within fences, maximum three attempts were made to drive an animal into nets to avoid the roe deer being excessively stressed. Handling upon capture lasted 3–5 min, and primarily involved sexing, approx. ageing (tooth wear) and collaring. The animals were blindfolded during handling. No sedation chemicals were used.

In Monte Bondone, net drives were deployed according to the general description, assuring a maximum distance of 15–20 m between operators on the drive line (according to the forest visibility), and 50 m between operators at the nets. Manipulation was undertaken as described for Rendena-Giudicarie area.

In Trois-Fontaines and Chizé, roe deer have been caught during annual winter captures since 1975 and 1977 respectively. Roe deer were caught using net drives between November and March. These operations require 150–200 people per day to drive deer into 3 km-long nets that are set each morning and afternoon procedure [71]. Once a deer was captured, it was removed from the net and transferred to a wooden retention box providing darkness and ventilation until the marking procedure. Trained and experienced operators then handled animals on a table in a shed. Marking and measurement procedures lasted for approximately 15 min. All animals were then released at the capture site.
