# Supplementary material for: Settle Down! Ranging Behaviour Responses of Roe Deer to Different Capture and Release Methods
Source: Animals (Basel). 2021 Nov 18;11(11):3299. doi: 10.3390/ani11113299 (PMC8614535; doi:10.3390/ani11113299)
Supplement: Supplementary file 1 [file animals-11-03299-s001.zip › animals-1421214-supplementary.pdf]

Supplementary 1 – Model selection to assess capture and release responses of roe deer in terms of their ranging behaviour.

Table S1. Model selection table for the distance to the centre of gravity of the locations after capture and release (GAM of the log-transformed distance with individual identity and study area as random effects).

Intercept.	Age	Capture method	Sex	Log Home range	Animal id	Study area	Spline of the time to capture by method	Age:method	Age:sex	Method:Sex	df	AICc	ΔAICc	AICc Weight
4.488				0.2842	+	+	+				512	222012.6	0.00	0.034
4.493			+	0.2850	+	+	+				512	222012.6	0.01	0.034
4.636	+		+	0.2829	+	+	+		+		512	222012.6	0.03	0.034
4.542				0.2832	+	+	+				512	222012.6	0.03	0.034
4.440		+		0.2832	+	+	+				512	222012.6	0.04	0.034
4.553	+		+	0.2843	+	+	+				512	222012.6	0.04	0.034
4.446		+	+	0.2838	+	+	+				512	222012.6	0.05	0.033
4.494	+	+		0.2825	+	+	+				512	222012.6	0.07	0.033
4.587	+	+	+	0.2825	+	+	+		+		512	222012.6	0.07	0.033
4.506	+	+	+	0.2834	+	+	+				512	222012.6	0.08	0.033
4.435		+	+	0.2839	+	+	+			+	512	222012.7	0.1	0.033
4.586	+	+	+	0.2825	+	+	+		+	+	512	222012.7	0.12	0.032
4.496	+	+	+	0.2835	+	+	+			+	512	222012.7	0.13	0.032
4.471	+	+		0.2738	+	+	+	+			512	222012.7	0.14	0.032
4.521	+	+	+	0.2814	+	+	+	+	+		512	222012.7	0.15	0.032
4.481	+	+	+	0.2745	+	+	+	+	+		512	222012.7	0.15	0.032
4.514	+	+	+	0.2812	+	+	+	+		+	512	222012.8	0.19	0.031
4.460	+	+	+	0.2747	+	+	+	+		+	513	222012.8	0.2	0.031
5.582					+	+	+				513	222013.3	0.76	0.023
5.578			+		+	+	+				513	222013.3	0.77	0.023

5.637	+			+	+	+				513	222013.3	0.78	0.023
5.623		+		+	+	+				513	222013.3	0.78	0.023
5.619		+	+	+	+	+				513	222013.4	0.79	0.023
5.636	+		+	+	+	+				513	222013.4	0.79	0.023
5.682	+	+		+	+	+				513	222013.4	0.8	0.023
5.682	+	+	+	+	+	+				513	222013.4	0.81	0.023
5.713	+		+	+	+	+		+		513	222013.4	0.81	0.023
5.612		+	+	+	+	+			+	513	222013.4	0.81	0.023
5.754	+	+	+	+	+	+		+		513	222013.4	0.83	0.023
5.677	+	+	+	+	+	+			+	513	222013.4	0.85	0.022
5.759	+	+	+	+	+	+		+	+	513	222013.4	0.87	0.022
5.587	+	+		+	+	+	+			513	222013.4	0.87	0.022
5.579	+	+	+	+	+	+	+			513	222013.4	0.88	0.022
5.661	+	+	+	+	+	+	+	+		513	222013.4	0.88	0.022
5.561	+	+	+	+	+	+	+		+	513	222013.5	0.92	0.022
5.656	+	+	+	+	+	+	+	+	+	513	222013.5	0.92	0.022

The “+” indicate variables included in the model.

Table S2. Model selection table for the distance between successive locations after first capture and release (GAM of the log-transformed distance with individual identity and study area as random effects).

Intercept	Age	Capture method	Sex	Log Home range	Animal id	Study area	Spline of the time to capture by method	Age: Method	Age:sex	Method: Sex	df	AICc	ΔAICc	AICc Weight
4.256				0.1312	+	+	+				473	348999.1	0.00	0.130
4.250			+	0.1304	+	+	+				473	348999.2	0.13	0.122
4.334	+	+		0.1277	+	+	+	+			476	349000.3	1.16	0.073
4.323	+	+	+	0.1271	+	+	+	+			476	349000.4	1.28	0.068
4.279	+	+		0.1330	+	+	+				477	349000.4	1.29	0.068
4.245	+	+	+	0.1320	+	+	+			+	476	349000.4	1.33	0.067
4.281	+	+	+	0.1323	+	+	+	+		+	475	349000.4	1.34	0.067
4.285		+		0.1323	+	+	+				478	349000.5	1.42	0.064
4.278		+	+	0.1323	+	+	+				478	349000.8	1.68	0.056
4.257		+	+	0.1319	+	+	+			+	478	349001.2	2.07	0.046
4.334	+	+	+	0.1267	+	+	+		+		478	349001.2	2.09	0.046
4.249	+	+	+	0.1267	+	+	+				479	349001.5	2.37	0.040
4.249	+			0.1322	+	+	+				479	349001.5	2.39	0.039
4.339	+	+	+	0.1276	+	+	+	+	+	+	479	349001.5	2.39	0.039
4.285	+		+	0.1328	+	+	+		+		480	349002.1	3.03	0.029
4.234	+		+	0.1358	+	+	+				483	349002.6	3.46	0.023
4.367	+	+	+	0.1278	+	+	+	+	+		481	349002.7	3.59	0.022

The “+” indicate variables included in the model.
